# Coexisting pelvic tuberculosis and endometriosis presenting in an infertile woman: Report of a rare case

**Published:** 2014-06

**Authors:** Maryam Eftekhar, Soheila Pourmasumi, Leila Motamed Zadeh

**Affiliations:** *Research and Clinical Center for Infertility, Shahid Sadughi University of Medical Sciences, Yazd, Iran.*

**Keywords:** *Tuberculosis*, *Endometriosis*, *Infertility*, *In vitro fertilization (IVF)*

## Abstract

**Background:** Primary and secondary infertility are the most common presenting symptom in patients with pelvic tuberculosis (PT). Endometriosis is commonly associated with an increased risk of infertility.

**Case: **Here, we report a rare case of coexisting PT and endometriosis in a 30-year- old woman, and the effects of controlled ovarian stimulation on reactivation of pathogen.

**Conclusion:** Coexisting endometriosis and tuberculosis of fallopian tube and ovary, as in present case, may alter clinical and radiological features, leading to difficulty in diagnosis. Early diagnosis with surgical exploration and adequate treatment can improve the chances of conception and also minimize morbidity.

## Introduction

Genital tuberculosis (GTB) mostly affects young women between 20 and 40 years of age and is relatively common in developing countries (1-2). Primary and secondary infertility is reported as the most common presenting symptom in GTB, followed by chronic pelvic pain, menstrual disturbances, vaginal discharge, pelvic masses and fever. However none of these are specific for GTB (3-7). Endometriosis defined as the growth of endometrial tissue outside of the uterus and is commonly associated with an increased risk of infertility (8-11).

It is surgically staged using the American Society of Reproductive Medicine staging system (12, 13). The mechanism for impaired fertility might be due to anatomic distortion from pelvic adhesions, endometriomas, and the production of substances (i.e. cytokines, prostanoids, and growth factors) which had adversary effects for normal ovulation, fertilization, and implantation (8-10, 12). Treatment options for infertility associated with endometriosis involve a combination of medical therapy, surgery, and assisted reproduction techniques (1, 3, 9, 14). Here, we report a rare case with genital tuberculosis and endometriosis coexisting in an infertile female, and the effects of controlled ovarian stimulation on reactivation of pathogen.

## Case report

A 30-year-old woman, gravida 0, with a 12-years history of primary infertility due to stage IV endometriosis admitted to Research and Clinical Center for Infertility, Shahid Sadughi University of Medical Sciences, Yazd, Iran. Primary diagnostic laparoscopy revealed bilateral tubal obstruction and severe pelvic adhesions. Despite adhesionolysis, she could not conceive. She also experienced 2 failed in vitro fertilization-embryo transfer (IVF-ET) attempts. Second-look laparoscopy revealed bilateral endometrioma and dense pelvic adhesions. The patient underwent to laparoscopically lysis of adhesions and bilateral cystectomy. In order to prepare for IVF procedure, the patient treated by a long gonadotropin releasing hormone agonist (GnRH-a) (Decapeptyl CR, 3.75 mg; Ferring, Malmo, Sweden) and human menopausal gonadotropin (Merional, IBSA, Lugano, Switzerland). In this case oocyte retrieval and in vitro fertilization was performed but embryo transfer was cancelled due to total fertilization failure.

Two months after ovarian puncture and discharge, the patient readmitted to hospital presenting with fever 39^o^C, acute lower abdominal pain, WBC and count of 13600. Transvaginal ultrasound showed complex right ovarian mass measuring 5x5 cm, with hyperechoic area. 

After that, optimal therapy consists of broad-spectrum antibiotics (i.e. ampicillin, gentamicin and metronidazole) was started. Because the patient experienced no clinical improvement, laparotomy was done and bad swelling pus (30 ml) detected, when the right ovarian puncture performed. Material culture showed no detectable bacterial growth. After relative symptomatic improvement, the patient was discharge from the hospital with oral antibiotics administration. The patient presented to hospital within eight months with fever and lower abdominal pain. Transvaginal ultrasonography revealed an echogenic fluid collection 3×3cm in the cul-de-sac. The patient did not improve by intravenous antibiotic therapy. The existence of pelvic tuberculosis was suspected by tuberculin skin test (PPD), so laparoscopic biopsy was taken and diagnosis of tuberculosis was confirmed according pathology finding. 

The patient was administered by a 6-months course treatment for TB, and received antituberclusis regimen containing isoniazid, rifampin, pyrazinamide, and ethambutol. The patient discharge after clinical improvement.

**Figure 1:**
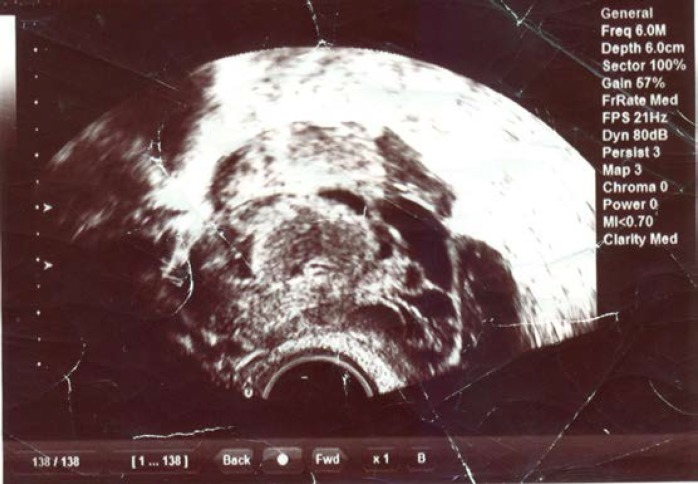
Transvaginal ultrasonography image

## Discussion

Pelvic tuberculosis (PT) infection is usually caused by reactivation of organisms from systemic distribution of *Mycobacterium tuberculosis* during primary infection (15-18). Pathogen is transmitted through hematogenous or lymphangitic spread, as well as direct extension from abdominal viscera (16-17). The clinical features are dependent on the spread of the disease. The fallopian tubes are the first and most commonly affected genital organs (90-100% of the cases) followed by endometrium (50-60% of cases), and ovary (20-30%cases) leading to a variety of clinical presentations such as chronic lower abdominal or pelvic pain, vaginal bleeding, menstrual irregularity, and infertility (4-7). However, tuberculosis pelvic disease may also create an adnexal mass, ascites or both and thus can be difficult to distinguish from other PID causes (19).

Endometriosis is found in 25-40% of infertile women, as compared to 2-5% of the general population (8-10, 14, 20). Although, endometriosis is proposed to be a risk factor for pelvic inflammation and abscess formation following transvaginal ovum retrieval (20). The evidence suggest that the presence of old blood in the endometrium provide a medium for bacterial growth after transvaginal ovum pick up (20). Nevertheless, IVF with embryo transfer found to be an effective treatment for infertility associated with endometriosis (19). Coexisting endometriosis and tuberculosis of tubes and ovaries may change clinical and radiological features, leading to difficulty in diagnosis, as seen in the present case (21). To definitively diagnosis, in our patient, we performed exploratory laparotomy that revealed tubo-ovarian masses containing 30 ml of bad swelling pus. 

In our case, coexisting endometriosis and tuberculosis was diagnosed postoperatively, on the basis of positive PPD test. The patient improved symptomatically with postoperative antitubercular therapy. Coexisting endometriosis and tuberculosis affecting both tubes and ovaries is very rare. Only one case was reported in 2008 by Himabindue *et al* (21). Such combined pathology has a greater impact on fertility and may lead to problem in diagnosis and treatment because of the unusual presentation. Early diagnosis by surgical exploration and adequate treatment may improve the chances of conception and also minimize morbidity. 
